# A global review of the impact on women from men’s alcohol drinking: the need for responding with a gendered lens

**DOI:** 10.1080/16549716.2024.2341522

**Published:** 2024-05-03

**Authors:** Ingrid M. Wilson, Bree Willoughby, Amany Tanyos, Kathryn Graham, Mary Walker, Anne-Marie Laslett, Leane Ramsoomar

**Affiliations:** a Health and Social Sciences Cluster, Singapore Institute of Technology, Singapore; b Judith Lumley Centre, Latrobe University, Melbourne, Australia; c Centre for Alcohol Policy Research, Latrobe University, Melbourne, Australia; d Institute of Mental Health Policy Research,Centre for Addiction and Mental Health, Toronto, Canada; e Dalla Lana School of Public Health, University of Toronto, Toronto, Canada; f Politics, Media and Philosophy, Latrobe University, Melbourne, Australia; g Gender and Health Research Unit, South African Medical Research Council, Pretoria, South Africa; h School of Health Systems and Public Health, University of Pretoria, Pretoria, South Africa

**Keywords:** Alcohol drinking, alcohol harms, partner violence, qualitative review, LMIC

## Abstract

**Background:**

Global evidence shows that men’s harmful alcohol use contributes to intimate partner violence (IPV) and other harms. Yet, interventions that target alcohol-related harms to women are scarce. Quantitative analyses demonstrate links with physical and verbal aggression; however, the specific harms to women from men’s drinking have not been well articulated, particularly from an international perspective.

**Aim:**

To document the breadth and nature of harms and impact of men’s drinking on women.

**Methods:**

A narrative review, using inductive analysis, was conducted of peer-reviewed qualitative studies that: (a) focused on alcohol (men’s drinking), (b) featured women as primary victims, (c) encompassed direct/indirect harms, and (d) explicitly featured alcohol in the qualitative results. Papers were selected following a non-time-limited systematic search of key scholarly databases.

**Results:**

Thirty papers were included in this review. The majority of studies were conducted in low- to middle-income countries. The harms in the studies were collated and organised under three main themes: (i) harmful alcohol-related actions by men (e.g. violence, sexual coercion, economic abuse), (ii) impact on women (e.g. physical and mental health harm, relationship functioning, social harm), and (iii) how partner alcohol use was framed by women in the studies.

**Conclusion:**

Men’s drinking results in a multitude of direct, indirect and hidden harms to women that are cumulative, intersecting and entrench women’s disempowerment. An explicit gendered lens is needed in prevention efforts to target men’s drinking and the impact on women, to improve health and social outcomes for women worldwide.

## Paper context

- **Main findings:** Women experience a multitude of direct, indirect and hidden harms from a male intimate partner’s alcohol drinking, particularly in LMIC settings.
- **Added knowledge:** This review consolidates global qualitative evidence from diverse women’s lived experience and adds a broader understanding of harm from men’s alcohol drinking, beyond physical and verbal abuse shown in quantitative evidence.
- **Global health impact for policy and action:** Policy and intervention efforts that take an explicit gendered and intersectional lens on men’s harmful drinking have potential to greatly improve health and social outcomes for women globally.

## Background

Harmful alcohol use is a leading population health risk factor contributing to the global burden of disease [1] with low- and middle-income countries (LMIC) carrying a disproportionate burden [2]. Globally, 18.4% of adults engage in heavy episodic alcohol use [3]. Accordingly, reducing alcohol consumption is a specific health target in the 2030 Sustainable Development Goals (SDGs) [4].

There is also growing recognition that alcohol misuse results in harms towards others and the gendered nature of this impact [5,6]. According to the World Health Organization (WHO) men in all societies drink more than women and men’s drinking causes more harm to others [2,7,8]. Additionally, women are disproportionately affected by the drinking of those close to them. Analysis across 10 countries found that 14% to 44% of women reported experiencing harm from a known drinker (i.e. a drinker who was not a stranger) during the previous 12 months; for women, the drinker was likely to be a man in a close proximity relationship [9]. Further, there is substantial evidence of alcohol’s role as a consistent risk factor in violence within the family and intimate relationships, and that it increases the severity of intimate partner violence (IPV) [10–12]. Accordingly, the WHO identified reducing alcohol-related harm as a factor in reaching other goals such as ending discrimination against women and girls (SDG 5–1) [4,13].

Despite this increasing awareness, reviews show that alcohol policy and interventions rarely focus on harms to women from men’s alcohol use [14,15]. In their rapid review of alcohol policy and harms to women and children, Karriker-Jaffe and colleagues (2023) [15] confirmed the small evidence base and argued that future policies and interventions need to include explicit attention to the impacts of men’s drinking on women. Current evidence is largely drawn from quantitative research focused on alcohol-related physical and verbal IPV and sexual aggression, and on measuring levels of risk and severity (see reviews based mostly on North American or European studies [16,17] and in Africa [18,19]). While these reviews expand the knowledge base in important ways, the diversity of effects of men’s drinking on women’s lives is less well-understood, especially from a global perspective.

The aim of this paper is to expand understanding of the broad impact of men’s drinking on women by reviewing the global qualitative literature. Qualitative research can provide a rich and nuanced understanding of the experience of alcohol-related harm, including inter-relationships of different impacts from men’s drinking, the process by which harms occur [20,21], and long-term impact on intimate relationships [22]. This review aims to contribute greater understanding of alcohol-related harm to inform the development of interventions that reflect the complexity of harm to women from men’s drinking.

The research question driving this review is: What are the harms experienced by women from men’s drinking and how do these harms impact women?

This narrative review is informed by a feminist ecological perspective that acknowledges that complex behaviours rarely have a single explanation and are influenced by an interplay of factors at various levels of the social ecology (e.g. individual, relationship, community, societal) [23]. No single factor explains why someone is violent or someone drinks in harmful ways, and the kinds of harms that result. These are influenced by individual traits, interpersonal relationships, community and social environments, and societal norms [24,25]. A feminist perspective further identifies the gendered nature of the harms from harmful alcohol use and the inequitable power dynamics in intimate relationships and in the broader social context that affect these harms [24]. A feminist ecological perspective also accounts for the harmful way in which men drink, often informed by social and gendered norms that place women at risk of alcohol-related harm.

While we acknowledge that harm to women from men’s drinking occurs in the LGBTQI population and extends to non-partner alcohol-related harms (e.g. rape, sexual assault, generalised violence), the present review focuses on harms from men’s drinking to women within heterosexual intimate relationships.

## Methods

### Design and search strategy

While systematic reviews aim to synthesise the best evidence on a narrow research question, we chose to conduct a narrative review due to the exploratory nature of our study goal. Such reviews are flexible and allow for evolving scope through the review process [26,27] and are ‘better suited to addressing a topic in wider ways’ [28]. We took an iterative approach: Phase 1 involved an initial exploration of the qualitative literature to scope the type and nature of men’s alcohol-related harms. Initial searches were conducted using Google Scholar and the Latrobe University library search engine. Next, we collated references identified from citations from papers in the original search and additional references known to research team. BW and AT conducted Title and Abstract screening and identified 80 papers of interest. These were then grouped according to type of harm addressed in the study (e.g. economic harm, sexual and reproductive harm, social harm) and distributed among investigators to identify papers meeting review criteria. Following team discussions, 20 papers were identified for inclusion, resulting in clearer inclusion criteria for a more comprehensive literature search.

Phase 2 involved a systematic search strategy using Medline, CINAHL, and EBSCOHost in October 2022. We used four concepts using the Boolean Operators: [alcohol/drinking] AND [harms] AND [wives/spouses] AND [qualitative]. The full search strategy is described in Appendix A. The search included peer-reviewed qualitative and mixed-methods studies published in English. We excluded literature reviews, theses, books and commentaries. No time limit was applied in order to be as inclusive and comprehensive as possible.

*Inclusion criteria*: Studies were selected if they:

- included a focus on alcohol (men’s drinking);
- featured women as the primary victims of alcohol-related harms;
- featured analysis of alcohol use explicitly in the qualitative results and discussion.

*Exclusion criteria*: Studies were excluded if:

- alcohol was mentioned only incidentally in women’s description of harms;
- focus was on the victim’s alcohol use;
- harms described were not directly linked to alcohol use.

### Study selection, data extraction and analysis

**Figure 1.**
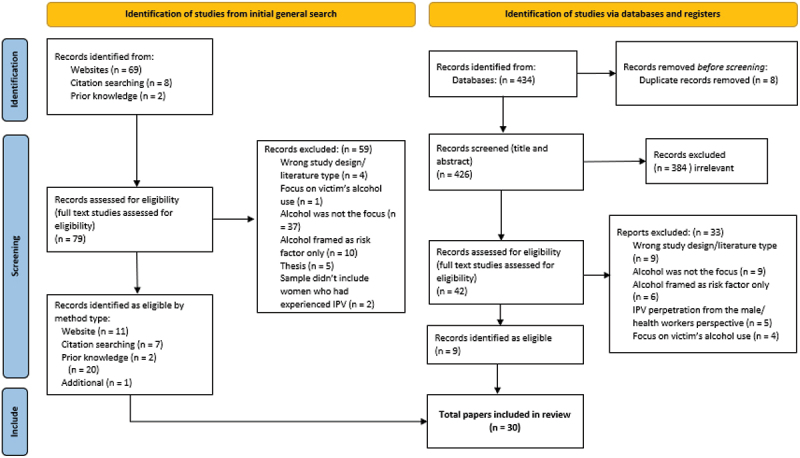
PRISMA flowchart.

Full text extraction of included studies was undertaken independently by all team members and then discussed within the team. Any disagreements were resolved through discussion with the whole team so that consensus was achieved on studies to be included. In total, 30 papers (28 studies) (see Figure 1). (Note. Our search generated one global systematic review of qualitative studies which featured 12 qualitative studies. As that review encompassed studies involving drug and/or alcohol use *and* IPV perpetration, we included only the cited studies that featured only alcohol use in the current review, though we note this is often combined with other substance use.) Consistent with guidelines for narrative reviews, we did not perform a formal quality review of each record [28].

## Analyses

IW and LR identified the different categories of harms described in the papers and organised these under the broader themes. We applied an inductive process to allow for emergent themes based on the observation of common patterns across the studies, for example, how participants described the role of alcohol in harm. The team then reviewed and discussed the themes. This process meets the SANRA standards (the scale for the quality assessment of narrative review articles) as applied to qualitative literature [28].

## Results

### Characteristics of included studies

The 30 papers are summarised in Appendix B. The studies were published between 1997 and 2022. Thirteen studies focused explicitly on alcohol use in connection with harm [21,22,29–37]. The remaining studies reported women’s experience of harm where alcohol use was described as a risk factor. Additionally, for two papers [38,39] that analysed harms connected to both alcohol and other drugs, only findings relating to alcohol use are discussed.

Four studies were conducted in low-income settings (Uganda [40–42], the Democratic Republic of Congo [43]); nine from lower-middle-income countries (India [30,36,44,45], Nepal [46,47], the Middle East (Lebanon and Egypt) [48], Ghana [49], Sri Lanka [50]); seven from upper-middle-income countries (South Africa [29,34,51,52], Colombia [53], Brazil [35], Thailand [31]); and ten from high-income countries (Australia [21,22,38,39,54], Denmark [33], Germany [55], Lithuania [37], the United Kingdom [32], USA [56]).

The majority of studies involved analysis of data collected through in-depth interviews or focus groups. The studies comprised a range of qualitative research approaches (grounded theory, ethnography and phenomenological research). Several basic descriptive qualitative studies used thematic analysis. Two papers from one study involved content analysis of transcripts of online counselling chats from partners of those with an alcohol or other drug problem [38,39].

Notably, many samples in the reviewed papers featured marginalised or disadvantaged populations, including women in Thailand living in refugee camps [31], populations in conflict-ridden DRC [43], and those in largely black townships in South Africa [29,34], rural communities in India [30,44,45], Aboriginal Australian women [54], Mexican immigrant survivors of abuse living in New York [56], young pregnant women living in urban slums in Kathmandu [47]; returned abductees from the civil war in Northern Uganda [40]; and low-income Middle Eastern families [48].

### Themes

We grouped the findings according to three themes: (1) actions taken by alcohol-affected men that resulted in harm to women (e.g. violent behaviour), (2) impact on women or harms experienced by women (e.g. injury); and (3) how participants *framed the role of alcohol* in their experience of harm from men’s drinking – a common finding across the studies.

**Figure 2.**
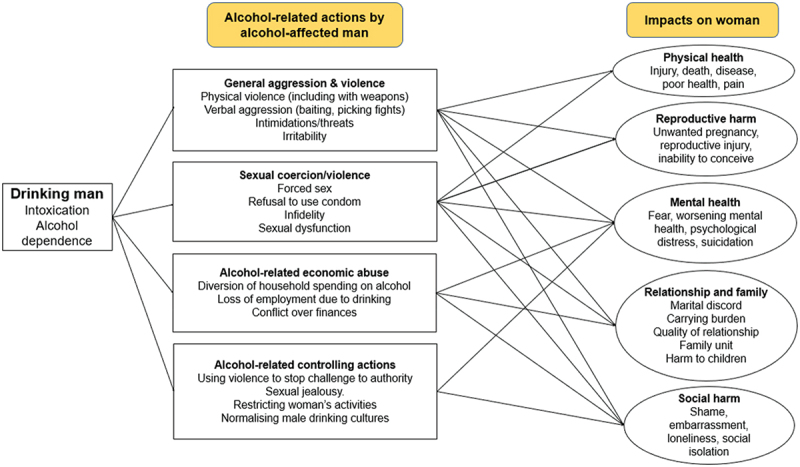
Schema of harms arising from men’s drinking and impacts on women.

Figure 2 lists the harmful actions and the harms experienced, while Table 1 shows the relevant papers. In the following, we describe specific harmful actions and impacts on women, representing women’s voices through selected quotes. Of note, the themes and the harmful actions and impacts described within the themes are overlapping and intersect.

**Table 1.** Themes and related studies.

Alcohol-related actions by alcohol-affected man		Impact on women	
Harms	Included Studies	Harms	Included studies
**General aggression and violence** (Physical violence; use of weapons; verbal aggression; psychological violence; intimidation; threats; irritability)	[21,22,29,34–37,39,40,44–46,48,49,51,52,56]	**Physical health** (Death; injury; disease; injury while pregnant; risk of HIV infection; STI infection; sleep problems, fatigue; pain) **Reproductive harm** (Unwanted pregnancy; loss of foetus; inability to conceive) **Mental health** (Fear; anxiety; psychological strain; lack of self-care)	[21,22,29,31,33,35,36,39,41–43,45–47,50–52,54,56]
**Sexual coercion/violence** (Forced sex; refusal to use condom; infidelity; sexual dysfunction)	[21,29,30,34,37,42,48,51,52,56]		
**Alcohol-related economic abuse and related behaviours** (Diversion of household spending on alcohol; loss of employment due to drinking; conflict over finances)	[21,22,29,35,42,43,52,56]	**Relationship and family** (Marital discord; carrying burden; quality of relationship; impact on family unit; harm to children; burden of caring)	[22,31,35–37,39,41,49,50,55]
**Alcohol-related controlling actions** (Reinforcing authority, jealousy; restricting women’s activities and isolating women; threat of violence; unsafe male drinking environments restricting women’s public movement; normalising male drinking cultures)	[21,22,29,34–36,42,43,48,52,53,56]	**Social harm** (Shame, embarrassment, humiliation, stigma arising from partner’s drinking, social isolation from avoiding drinking situations)	[21,22,31,33–37,42,43,50,55,56]

### Alcohol-related actions by the alcohol-affected man

#### General aggression and violence

Studies of alcohol’s role in violence towards women described a range of harmful acts from moderate to severe. We note that the specific role of alcohol was not always clear in the descriptions, especially in contexts where abuse of women occurred, regardless of alcohol consumption.

##### Physical violence

Women’s descriptions revealed that they were subject to severe forms of alcohol-related physical violence, including hitting, beating with fists and/or other objects, shoving, pushing downstairs, pulling hair, threats with weapons, burning, and throwing a woman off a balcony [21,34,36,40,46–48], with ‘beating’ the most common term used. Women also described being punched or kicked in the abdomen while pregnant [37,47]. One study involving 18 Australian women [21] categorised alcohol-related physical abuse as “severe (e.g. choking, beating, hitting, kicking, punching, dragging by the hair, eye gouging, twisted/broken fingers) and moderate (e.g. pushing, slapping, shoving, grabbing, pushing up against wall, throwing things at her that could hurt” [21] (p. 119).

A young pregnant woman living in urban slums in Kathmandu described devastating violence by her partner:

Since that day, he (husband) started drinking in unlimited amounts. He comes home at midnight and beats me with such a big stick (showing the size with her hand). I don’t even remember the number of times he beat me because he beats me till he cools down and blame my maternal home for giving birth to me. [47]

##### Verbal aggression

Women reported being on the receiving end of alcohol-related verbal abuse, e.g. cursing and bullying [34,48], scolding [36], yelling, screaming, shouting, intimidating, ‘in your face’ [21]. Commonly alcohol-related emotional abuse included insulting, belittling, humiliating in private or public (e.g. calling names, degrading comments) and treating the woman with contempt and disrespect [21,37].

##### Intimidation/threats

Studies revealed that women (and children) experienced alcohol-related intimidation that instilled fear [29,46] such as threats to kill or hurt her or someone whom she cares about, and damaging property (e.g. punching holes in the wall) [21]. Women also described fear and their avoidance strategies relating to a partner’s irritability when coming down from a big drinking session [21,49].

#### Alcohol-related sexual aggression and coercion

Several studies emphasised the role of men’s alcohol use in sexual aggression [21,29,30,34,37,42,48,49,51,56] with partner’s alcohol use directly implicated in sexual violence and coercion. One Aboriginal Australian woman described the impacts of her husband’s sexual violence:

One of the incidences I just stepped out of the car while it was driving, I have taken overdoses through the situation, I have tried to commit suicide. I just couldn’t take it no more. When he has had alcohol, he is particularly violent during sex, yeah, that’s right. Sometimes I fight back, not always. He is very strong, and I don’t really have a chance, have I? [54]

Male participants also acknowledged alcohol as a contributing factor in sexual coercion. In a study of Ugandan men and women’s perspectives on sexual violence and HIV/AIDS, around 65% of men (an estimated 88 of 133 men who admitted to forced sex with their wives) indicated that alcohol was a contributing factor [42]. Women’s experiences of forced sex when the partner was drunk included during culturally prohibited times such as menstruation and following childbirth [42]. Further, men were less likely to agree to condom use when drinking, thereby reducing women’s ability to negotiate safe sex or refuse sex and elevating their risk of HIV, STIs and unwanted pregnancy.

He drinks and comes home very late at night. … and when he is drunk, he does not want to use a condom. [51]

#### Alcohol-related economic abuse and related behaviours

The economic impact of men’s drinking on women, and by extension on the household, was also common. Women often reported the diversion of money intended for household spending (e.g. food, clothing and medicine) towards expenditure on alcohol [21,42]. They spoke of being threatened or coerced to hand over their earnings or even to buy alcohol for their partner. In this way, men’s drinking contributed to a loss of resources for the household, and overall household financial instability [43]. Resistance by women relating to financial issues often led to conflict and argument and further violence by the alcohol-affected man [21].

My husband drinks all the time. Whenever I ask him to stop drinking, he curses and beats me. One time, he took his whole salary and spent it on drinking and didn’t give me any money for food. I warned him that drinking will make him do worse things than losing money and then he beat me. [48]

It interferes because he would rather spend his money on alcohol than spending with the children. Drink is the priority, we sometimes lack food, but not drink … [35]

Men were commonly seen as abdicating their ‘traditional role as provider’ through loss of employment or missed work due to drinking [35], being unable to provide children with basic necessities [29,35,56], so that responsibility for supporting the family defaulted to women [21] whose income potential was typically much less than that of men. Thus, men’s drinking (particularly in low-income settings) placed women in precarious financial positions; in cases of extreme economic deprivation and poverty, women reported being forced into engaging in ‘survival sex’ (prostitution, sex trading) to support alcohol-affected households [52].

#### Alcohol-related controlling actions

For some participants, the pattern of alcohol-related abuse operated as a means of exerting control over the female partner. In a study of low-income female victims in Lebanon and Egypt, participants reported that beatings would often occur when women’s disapproving words or actions were perceived as challenging their husbands’ authority, with alcohol increasing the perception of authority challenge – ‘the drinking simply exacerbated other perceived violations of responsibility on part of the wife which led to violence’ [48].

Sexual jealousy, a common manifestation of patriarchal control, was reported to be heightened when the partner was drunk [21,22,53].

It was predictable that … we weren’t going to have those same issues [his jealousy] if he wasn’t drinking. They were … gonna come about … if it was a big night out with the boys and he had been drinking … (Simone, 28 years). [22]

Alcohol-related abuse was presented as part of controlling behaviours such as restricting women’s involvement in economic decision-making, access to resources, mobility, work and socialising [21]. Some women reported not being allowed to have a car, study, or socialise outside of the home [21,35,47]. Fear of violence from an alcohol-affected partner also operated as a tool of control of women. In one study an Indian woman compared having an alcoholic husband to ‘being in jail’ because of the husband’s alcohol-related violence:

You cannot do anything freely because anything you do may provoke your husband into beating you. [45]

Several studies pointed to the male drinking culture; for example, alcohol consumption was identified as essential to Ugandan culture and an important vehicle for male socialising [42] and male-dominated environments were often unsafe for women in South Africa [52]. Thus, male drinking cultures of intoxication and violence served to constrain women’s movement and access to public space [30].

### Impacts on women

#### Physical, reproductive and mental health harms

In addition to physical health harms such as injury and death from alcohol-related IPV, the review highlighted reproductive harms experienced by women including: unwanted pregnancy, reproductive injury, inability to conceive and child deaths caused by alcohol-related violence during pregnancy [29].

… .I was sleeping, my husband came home drunk at midnight. He asked me to sleep with him, but I denied … .I told him we should not do it because I am seven months pregnant. He then kicked me on my abdomen and slapped me on my cheeks … … Then he pulled my hair and pushed me down the stairs. [47]

In several studies, women claimed that alcohol contributed to their husband/partners’ infidelity and risky sexual activity, increasing women’s risk of HIV or other STIs [29,42,51,56].

Studies described how men’s drinking led to long-term physical effects for women, including fatigue, anxiety, sleeping problems, body aches and pains, and losing weight [36]:

I have taken [a] controlled drug since I was 22. I started taking it because I got sick. Nervous, I could not sleep when he got home drunk, screaming, swearing … [35]

The impact of alcohol-related actions on women’s mental health was identified in many studies. Women reported psychological distress, self-harm, depression and suicidality, particularly in response to experiencing sexual violence [54]. One study in Sri Lanka reported incidents of self-harm as a cumulative effect from the stressors from men’s drinking that aggravated non-alcohol-related life stresses [50]. Women also reported anxiety arising from the need for constant vigilance in facing the partner’s unpredictable behaviour [21,33,39] or threats of violence [35,41] when drinking or recovering after a drinking episode [21].

He promises to strangle my neck and this scares me and leaves me unstable and worried. [41]

… he would have no idea why I would be a nervous wreck or miserable or an absolute basket case. [21]

In other studies, women described feelings of fear and anxiety regarding their children’s safety when their partner was alcohol-affected [22,33], concern for the future, suicide ideation and loss of hope for the future [36,54]. Persistent fear by women due to their partners’ alcohol use was associated with a lack of self-care and care of their children [31,43].

Analysis of online helpline chat scripts from women with partners with alcohol and other drug (AOD) issues revealed a pervading sense of sadness and despair, highlighting the impact of men’s drinking on all aspects of women’s lives [38,39]. The experience of drunken verbal abuse from a partner also has an impact on women’s self-esteem and identity:

When I’m alone like now I just feel these waves of despair and utter helplessness (female, 20–24 years). [39]

I also find myself believing some of his abusive names such as “idiot” and “useless” (female, 40–44 years). [39]

#### Harms to the intimate relationship and family functioning

A common finding was the contribution of men’s drinking to marital discord and family dysfunction [22,35,37,39,49,50]. Participants described how a husband’s intoxication contributed to more verbal aggression and fights, with drunkenness becoming the catalyst leading to arguments becoming violent [45] with corresponding impacts on family functioning.

Men’s drinking and its impact on the household and family functioning appeared as a central focus of conflict and arguments with women reporting being intimidated and threatened with violence if they questioned or quarrelled with their husband/partner about his drinking or drunkenness [46].

The quality of intimate relationships was affected by men’s drinking in several ways. As noted, alcohol was viewed as culpable in men’s infidelity [29,42,51,56], and men’s drunken jealousy resulted in accusations of female infidelity [21,22,53]. A Ugandan study reported that partner alcohol use contributed to sexual dysfunction affecting the intimate relationship [42]. Women also reported loneliness, especially during pregnancy, because of exclusion from socialising at drinking occasions [55]. The avoidance of drinking husbands as a protective measure also created emotional distance in intimate relationships over time [22]. Women also reported anxiety and ambivalence about the future of the relationship with a confluence of emotions of fear, hope, and love [39].

Men’s drinking affected families in a range of ways beyond the partner relationship. As an example of the impact on limited household economic resources, one study of participants in a refugee camp in Thailand described children going to school with empty stomachs [31]. Studies also reported the effect of men’s alcohol use on quality family time and communication; men were reported to withdraw from social and family relationships and display irritability with family members [22,36,39,49]. Husbands’ loss of employment due to drinking and neglect of family responsibilities meant that women had to assume the burden of household responsibilities, earning and parenting.

I look after the kids on my own 7 days a week, no break … his days off he doesn’t help he just goes out, gets drunk (female, 30–34 years, alcohol). [39]

Studies also highlighted the impact of men’s drinking on the children [22,35]. Women feared for the safety of children who were sometimes also targets of physical and verbal abuse from their father [37], faced neglect and loss of a father figure [35], and suffered indirect harm from witnessing alcohol-related violence [37].

Each night we walk on eggshells as we never know when the mood will change, but when it does he usually puts the children and I down in a verbal sense (female, 40–44 years, alcohol). [39]

#### Social harm – shame, loneliness and isolation

Participants spoke of feelings of shame, humiliation, embarrassment, loneliness, and social isolation connected to their partner/husbands’ drinking [22,31,35–37,42,43,50,55]. In one study, women expressed feelings of shame from forced sex coupled with knowledge that their drunk husbands would seek sexual relationships outside the marriage [42]. Others experienced social harm arising from husbands creating disturbance at their workplace [34] or taking responsibility for his care in public situations:

I really hate what alcohol does to him. We would fight at home; the next thing he shows up at my school drunk and demands that we talk about our fight right there. He embarrasses me at my workplace. [34]

Some women reported avoiding social situations due to humiliation they anticipated from their husband’s heavy drinking [22,36]. Even after ending the relationship with an alcohol-affected partner, some women evaded social situations involving alcohol to avoid being reminded of experiences of alcohol-related abuse.

I get sort of a trauma reaction if people were drinking too much around me, so I don’t tend to socialise much in that area. (Anne-Marie, 45 years) [22]

Studies revealed the loneliness and isolation from partners’ alcohol-related absences, but also being kept away from friends, family and a wider social network, due to his drinking [33]. Women with alcoholic partners in Brazil reported a loss of freedom, and not being able to do things they wanted [35]. Danish women noted the harmful impact on their social lives of not being able to invite friends to their home and not feeling comfortable socialising with their partner [22,33]. Some women also lamented a lack of companionship, reporting increasingly leading separate lives from the alcohol-affected partner [22,37]. Difficulties in disclosing their experience also contributed to isolation [33] and its ongoing impact:

This isolation is also because you comfort yourself once, twice, six times, 106 times, and you realize, how much longer can you cry about the same thing? (Female, age 55) [37]

The sense of despair and hopelessness expressed by women in several studies stemmed from the inability to effect change in their circumstances due in part to their partner/husband’s lack of recognition of a problem and unwillingness to seek help, as well as the impact of cultures that normalise men’s drinking [22].

Nearly all of the participants whose partners drank heavily viewed alcohol treatment as a solution for the abuse behavior. Yet, most partners expressed reluctance to seek treatment, arguing that it was normal for Mexican men to have a few drinks after work or with their friends on the weekends. [56]

### Framing the role of alcohol

How studies framed the role of alcohol helps understanding of the broader harms and impact of men’s drinking on their female partner. Studies (and study participants) consistently framed alcohol as a main cause of, and inextricably linked to, the perpetration of IPV and other harms [21,32,34,41,43,44,46,48]. Almost 100% of the married women interviewed for a study in rural India blamed their husband’s violence on alcohol [44]. In another study, a young pregnant Nepalese woman stated *‘It’s only because of alcohol. There are no other reasons’* [46]. South African survivors of domestic violence described alcohol as a force outside of the man, the ‘demon of alcoholism’ that must be cast out [34].

Alcohol was also often described as a *trigger* for conflict and arguments becoming violent [45] with violence routinely linked to alcohol use [40,46,47], although one study observed that no consistent pattern emerged between husband’s level of intoxication and severity of violence [56], and some women in an Indian study acknowledged that violence sometimes occurred when their husband was sober [44]. In some contexts, where physical and other forms of violence towards women are common, alcohol was seen as exacerbating rather than causing the violence [45].

In several studies, participants presented a duality of the drinking man – the ‘good man’ sober, versus the ‘bad man’ drunk [21,33,34,41].

He only beats me when he is drunk. That is when he has the strength to fight. But when he isn’t drunk he is good. [41]

My husband beats me whenever he is drunk. He is not always drunk, though. He is a nice person when he is sober. [34]

However, participants in some studies questioned an unassailable causal link between alcohol and violence. Satyanarayana et al. (2015) [36] observed in their study in India:

Few women strongly opposed the idea that alcohol consumption alone was the cause of violence. One participant said if he is so intoxicated and lacks awareness of what he may be doing, why does he beat only his wife and children? He beats us because he can and we take it … there are other people in our house as well, why doesn’t he beat them? [36] (p. 40)

Similarly, domestic violence survivors in a UK study acknowledged alcohol’s role in their husband’s violence, however some questioned men’s appealing to alcohol as an excuse:

I think that whatever they do when they’re drunk it may be that they wanted to do it anyway, the drink’s just let them do it. [32]

Some participants remarked on the temporal pattern of violence co-occurring with drinking, with men peaceful during the week but violent at week’s end, when a paycheck allowed purchase of alcohol [29,54]. In an Australian study, participants described a cycle of drinking and violence, where men were happy and fun upon starting to drink, with aggression and targeted violence towards a partner escalating with intoxication. Violence was not always the outcome, but women in this and other studies recognised the pattern and experienced anxiety whenever their male partner drank. Women adopted preventive and protective strategies according to recognisable stages of his drinking [21,34]:

I never argued with him, especially when he is drunk. I know he is not responsible for his actions. Some forces of darkness make him behave that way. I sometimes pretend to be asleep when he comes home very late. I will just sleep with him to avoid noise. [34]

## Discussion

The aim of this review of qualitative research was to explore and document the breadth, nature and impact of the alcohol-related harms perpetrated by men towards women. The studies described harms experienced when the partner is alcohol-affected as well as those that were a consequence of his persistent alcohol use. Harms and impacts were often hidden; the impact on women is either not seen, or if seen, is not always recognised as harm, and women consequently do not get the help, support or services they need.

Our review identified and categorised multiple types of harmful actions by men related to their alcohol use. Women’s descriptions of severe violence from an alcohol-affected partner revealed how alcohol adds to the volatility of the violence they face, reinforcing quantitative evidence linking alcohol use and IPV severity [57,58]. Women’s significant vulnerability to sexual coercion and violence when their partner was drunk was a consistent finding across studies. While there is a growing research focus on sexual violence in intimate relationships [59], the role of alcohol has received little attention, despite considerable research on alcohol’s role in perpetration and victimisation of *non-partner* sexual assault [16]. Further attention in prevention efforts to the facilitative role of men’s drinking in *intimate partner* sexual aggression is needed.

Significantly, this review highlighted the far-reaching and often insidious impact of men’s drinking on women’s lives. In addition to acute and chronic physical injuries from violence, women’s descriptions revealed how men’s drinking affected their intimate relationship and family functioning, exposed women to mental health harm, and increased their social isolation. The review further highlights the economic hardship stemming from men’s drinking and affecting women, largely related to men’s control of resources and the diversion of household finances to his alcohol use. Economic abuse is gaining recognition as a form of IPV, yet research has as yet largely ignored men’s harmful alcohol use as a form of economic abuse [60]. This review reveals how men’s drinking can operate to further entrench women’s economic disempowerment, especially in lower-income countries or disadvantaged populations

The framing of alcohol as a direct contributor, trigger or cause of men’s violence and aggression is consistent with literature on victims’ attributions for IPV [61]. It was clear from reports by female participants from some countries that intoxication both explained and excused men’s violence, although participants in some studies rejected this excuse. There has been a long-standing debate about alcohol as a cause of men’s violence towards women, and there remain widely held norms that perpetuate the excuse value of alcohol [62,63]. Interventions to reduce alcohol-related harm to women must focus on men’s accountability for their drinking and harm. And regardless of the framing, support services need to engage with women’s lived experience to better understand the significant role of men’s drinking in harm, which is unambiguous from this review.

Low- and middle-income countries carry a disproportionate burden of harmful alcohol use [2] which may explain the preponderance of studies from these contexts. Studies highlighted multiple and significant disadvantages for women harmed by men’s drinking in these countries. Future studies and interventions need to tailor prevention and response programmes to these heterogeneous contexts and acknowledge the intersectionality of the problems of alcohol use, harms to women and poverty often found in LMIC contexts.

From a feminist ecological perspective, harmful alcohol use and harm to women occurs within a gendered context that is underpinned by gender inequality. In many of the reviewed papers, men’s drinking and heavy drinking were normative. Heavy drinking is well-established as performative of (hegemonic) masculinity in public [64] and in private, as in alcohol-related IPV [65,66]. A large body of evidence across HIC and LMIC links men’s alcohol use to social norms of masculinity, symbolising toughness and dominance, and as way of affirming they are ‘real men’ [67,68]. In our review, women reported that challenging their partner’s drinking often resulted in conflict and arguments that escalated to violence. In this way, men’s entitlement to drink appeared inviolable, even in the face of the significant harms it caused to others. Hence, interventions that only target drinking at the individual level will be limited. Research and policy on alcohol-related harm to women needs to explicitly target the culture of men’s drinking that enables such harms to persist.

## Implications for research, policy and prevention

Addressing alcohol-related harm to women is crucial to meeting the Sustainable Development Goals. As noted, policy interventions that address the harms to women from men’s drinking are scarce [14,15], and despite overwhelming evidence of men’s drinking causing harms, alcohol policy interventions are largely de-gendered, and avoid targeting responses to men [69]. To fill this gap, alcohol policy scholars have proposed new conceptual models to improve policy responses to alcohol-related harm to women (and children) that holistically consider drinking norms, social norms around masculinity and power, gender roles and gender inequality, and that encompass broader harms beyond physical and psychological harms [15]. By presenting a detailed picture of the broad nature of alcohol-related actions and impact on women, our review findings can help to inform the development of interventions that specifically target men’s drinking, and the corresponding harms experienced by women.

Based on the outcomes of this review, we recommend the following:

- Research, policy making and intervention design that adopts an explicit gendered lens. Work aiming to address harmful alcohol use and harm to others needs to explicitly consider the needs of people of all genders in the planning, analysing, designing and decision-making. Applied to the findings of this review, gender should be included as a category of analysis in understanding alcohol-related harm, and efforts should target men’s drinking and the specific nature of the harms to women. We need to give careful consideration to the needs of women, and draw on methods that help to achieve that goal (e.g. feminist research methods);
- Further quantitative research to measure and document the full range of harms associated with men’s drinking, and the type and extent of harms on women. Our review highlights several harms that are currently missed or under-estimated, especially by quantitative studies (e.g., financial and social harms);
- More qualitative research to better understand the relational dynamics for women living with a partner with alcohol problems, and that explores men’s perspectives on their own drinking and harm to others;
- Further investigation of alcohol’s role in sexual aggression within intimate relationships, and men’s harmful alcohol use as a category of economic abuse;
- More cross-cultural studies and culturally and contextually relevant research and policy making to acknowledge the intersectionality of alcohol use, harms to women and poverty. The specific nature of harms to women in poorer, patriarchal countries are often overlooked (for example, women needing to turn to prostitution to combat the loss of household income from men’s drinking);
- Research to further explore attitudes towards alcohol use and harm to women and interventions to challenge or disrupt the excuse value of alcohol; and
- The inclusion of more participatory methods that amplify the voices of diverse women with lived experience in research, policy and interventions and service development.

## Strengths and limitations of this review

By synthesising global qualitative research, this review contributes a nuanced, broad and in-depth understanding of the impact of men’s drinking on female intimate partners. The preponderance of research studies from low resource settings countries is a limitation in terms of generalisability, but also an advantage as these countries tend to be under-represented in most research. These studies also provide important insights on how alcohol use may intersect with gender norms within different sociodemographic and cultural contexts. Our search was limited to peer-reviewed, English-language publications and hence may have missed additional perspectives captured in grey literature and other sources. A further limitation is the focus only on heterosexual relationships in order to address heteronormative gendered dynamics relating men’s drinking to harms to women. Research exploring alcohol and harms in LGBTQI+ intimate relationships is warranted.

## Conclusions

Men’s harmful alcohol use causes a multitude of direct, indirect and often hidden harms to women. Our global review of qualitative research describes the many harmful acts towards women related to men’s drinking and the range of impacts on women, including physical injuries, death, loneliness and isolation, and many others. These different harms intersect and have a cumulative and cascading effect, adding to women’s disempowerment, potentially increasing their vulnerability to further harms. The review points to a range of ways that men’s drinking reduces women’s autonomy; repeated patterns of intoxicated violence (or threat thereof) controls women through fear; alcohol-affected sexual demands and unprotected sex reduces women’s reproductive autonomy; alcohol-related financial abuse reduces women’s already diminished economic power, and increases her caring burden. It is important to recognise that men’s drinking sits within a gendered context. Understanding the complexity and diversity of the intersection of men’s alcohol misuse, the impact on women and the cultural context is key to prevention planning and service response.

## Appendix A: Search strategy

**Table:**

Search ID#	Search Terms	Search Notes
S1	exp Alcohol Drinking/	Subject Heading (MeSH)
S2	exp alcoholism/	Subject Heading (MeSH)
S3	‘alcohol use’ or ‘alcohol abuse’ or ‘binge drinking’ or alcoholism or ‘binge alcohol consumption’ or ‘alcohol* drink*’ or ‘alcohol misuse’ or ‘harmful alcohol use’ or ‘alcohol use disorder’ or ‘alcohol* intoxicat*’ or intoxicat* or ethanol or ‘ethanol use’ or ‘ethanol abuse’ or ‘risky alcohol use’ or ‘hazardous drink’ or ‘alcohol addiction’ or ‘alcohol dependen*’ or ‘problem* drink*’ or ‘risky* drink*’ or ‘high risk drink*’ or ‘alcohol consumption’ or ‘alcohol drink* habit*’ or ‘alcohol intake*’ or ‘alcohol drink* attitudes’ or drunkenness* or family stress coping	Keyword
S4	S1 or S2 or S3	Combination OR
S5	exp Intimate Partner Violence/	Subject Heading (MeSH)
S6	exp Domestic Violence/	Subject Heading (MeSH)
S7	‘intimate partner violence’ or ipv or ‘domestic violence’ or ‘domestic abuse’ or ‘spous* abuse*’ or ‘family violence’ or ‘violence against wom?n’ or ‘intimate partner abuse’ or ‘partner abuse’ or ‘wife abuse’ or ‘abused wom?n’ or ‘battered wom?n’ or ‘battered female*’ or ‘wife beating’ or ‘battered wives’ or ‘battered wife’ or ‘domestic assault’ or ‘marital abuse’ or ‘partner aggression’ or ‘partner assault’ or ‘dating violence’ or ‘sexual* abus$*’ or ‘sexual* aggress*’ or ‘sexual* assault’ or rape or fear or ‘physical* violen*’ or ‘physical* assault’ or ‘physical* abus*’ or ‘physical* aggress*’ or ‘emotional* abus*’ or ‘psychological* abus*’ or ‘psychological* aggress*’ or ‘coercive control’ or ‘control* behavio?r’ or ‘spous* assault’ or ‘husband abuse’ or ‘husband aggress*’ or ‘intimate terrorism’ or ‘common couple violenc*’ or ‘situational couple violence*’ or ‘violent resistance’ or ‘intimate homicide’ or ‘domestic homicide’ or conflict or argument* or femicide	Keyword
S8	S5 or S6 or S7	Combination OR
S9	Wives of alcoholics or spouse* of alcoholics or family	Keywords
S10	qualitative study or qualitative ethnograph* or narrative account or participant observation or grounded theory or interpretative or phenomenological analysis or case stud* or focus group or thematic analysis or framework analysis or framework approach in-depth interview or semi-structured interview or mixed-method*	Keywords
S11	S4 AND S8 AND S9 AND S10	Combination AND
Limiters:	1. Peer reviewed 2. English language 3. Age: adult (19–44) & middle age (45–64) … used in CINAHL only	Used only on CINAHL as Medline database includes peer-reviewed studies

## Appendix B: Summary of included studies

**Table:**

Author	Country	Study aim	Method and study approach	Sample and setting	Key findings (related to alcohol)
Annan & Brier [40]	Uganda	To describe the experience of women who return from abduction in Northern Uganda, and how violence from an armed group interacts with family violence, poverty, and structural factors, including IPV.	Grounded theory	21 females who returned from abduction in Northern Uganda	Alcohol use was a cause of conflict and exacerbated tension caused by scarce resources. Public drinking and intoxication were common. Brewing of alcohol as a main source of work placed single women who had no male protection in the home at risk. Alcohol was stated as a problem in every case of IPV. Participants reported aggression and physical abuse related to partners’ heavy drinking and intoxication; arguments over resources; alcohol-related conflict between family members and abuse, verbal abuse and shaming from (drunk) mother-in-law
Backe et al. [29]	South Africa	To examine relationship of alcohol and IPV in Soweto townships	Ethnography, life history narratives	77 women in Soweto, Johannesburg, South Africa	Alcohol use by an intimate partner, or an abusive family member, was a consistent through-line in women’s testimonies, sometimes as a contributing factor to physical violence and infidelity and, in other instances, a habit that depleted the financial resources of families, constituting a form of economic abuse. One woman reported two child deaths which she attributed to alcohol-related violence during pregnancies. 70% of those experiencing IPV attributed violence to the man’s alcohol use (referred to as coercive violence). Several women witnessed IPV and alcohol-related IPV stemming from father’s drinking as children.
Bloom et al. [41]	Uganda	To examine intimate partner violence among Ugandan women living with HIV, their experiences of disclosing such violence and how culturally normative factors affected disclosure-related outcomes.	Sequential explanatory mixed-methods study; thematic analysis of qualitative component	Sample of 168 Ugandan women living with HIV and with experience of IPV completed survey; 14 in-depth interviews	Alcohol use by an intimate partner was a significant contributor to women’s experience of IPV and barrier to disclosure. Two main themes from interviews: (1) women who disclosed IPV were advised to ‘endure’ the violence; (2) alcohol ‘excuses’ violence from a partner with the focus on drinking as the culprit, not the man.
Cash [42]	Uganda	To understand experiences and interpretations of the relationship between sexual violence and transmission of HIV AIDS	Qualitative (ethnographic focus group discussions and In-depth interviews)	450 Ugandan men and women	Men’s alcohol use contributed to infidelity, forced and unprotected sex and domestic violence. Women reported experiencing forced sex frequently during menstruation, and following childbirth. Approximately 65% of men who had forced sex with their wives, said alcohol was a contributing factor. Financial harms were experienced by the women and the household due to money spent on alcohol. Alcohol contributed to sexual risk behaviour (no condom, multiple partners, transactional sex) and women felt shame when their (drunk) husbands sought sexual relationships outside the marriage. 86% of male respondents viewed alcohol as a means of socialising with other men and essential to Ugandan culture.
Chowdhury et al. [30].	India	To determine how social changes in the region influence changing patterns of acceptable and problem use of alcohol.	Ethnographic study using participant observation and focus group discussions	Average of 9–10 group interviews (single-sex/mixed-sex) per village and a total of 88 in-depth interviews were conducted in Sagar and Gosaba Blocks of the Sundarban Region	Drinking was observed to be an integral feature of the cultural landscape. Effects of problem drinking included: social disturbances, family discord, and domestic violence. Men’s drinking was the cause of marital and family conflict; men controlled earnings and spending and the cost of drinking affected domestic finances. Men also demanded higher dowries. Men’s drinking behaviour also made them less willing to work, leading to economic distress, family conflicts, and criminality. Women experienced physical and mental violence, sometimes fatal, and shame due to experiencing violence. Women’s movement was constrained due to men being drunk in public spaces.
Clark et al. [46]	Nepal	To examine the prevalence and risk and protective factors for physical/sexual IPV within marriages	Mixed methods, but qualitative component included case-based analysis	36 (18 women and 18 men) couples from 3 districts in Nepal	Wives/partners reported experiencing physical violence, intimidation and threats of violence from a drunk partner, particularly if they questioned or quarreled with their husband/partner about his drinking or drunkenness. They reported feeling fearful of their husband/partner when he threatened violence when drunk.
Deuba et al. [47]	Nepal	To better understand the IPV experienced by young pregnant women in urban slums of the Kathmandu Valley, and identify their coping strategies, care and support seeking behaviours.	Qualitative content analysis	20 pregnant women aged 15–24 years from 13 urban slums in the Kathmandu valley	Fourteen women were survivors of violence. Husband’s heavy use of alcohol was linked to IPV. Women reported that they were more likely to experience violence if a husband had an alcohol use disorder. Husband coming home intoxicated was one of five major reasons given for physical and psychological violence.
Ezard [31]	Thailand	To better understand the use and role of alcohol within a Mae La refugee camp in Thailand.	Qualitative interviews	97 participants aged 15 years + (Karen men and women from Myanmar) living in a refugee camp	IPV emerged as a prominent theme with four sub-themes: ‘Alcohol is Happy Water’ (for men); alcohol use is changing in displacement; IPV is an emergent alcohol-related harm; and the relationship between IPV and alcohol is complex. Alcohol use is subject to strongly gendered social controls. Partners’ excessive alcohol use led to failure to contribute to household resources, selling or exchanging rations and possessions as a source of income for alcohol; children going to school with empty stomachs; and women’s depression (affecting her ability to look after children properly).
Fox et al. [51]	South Africa	To examine the intersections of risk for intimate partner violence and HIV infection in South Africa.	Qualitative descriptive study. Semi-structured interviews	18 women (aged 18–53 years) seeking services for relationship violence	Women reported that substance use, particularly alcohol, was related to their experience of physical, sexual and emotional abuse. Common pattern of partner arriving home drunk, rapidly becoming violent and demanding sex. Women reported that intoxication made men less likely to agree to condom use and more likely to have sex outside the relationship, increasing STI risk for women; alcohol could increase unwanted pregnancies and infidelity.
Galvani [32]	UK	To explore women’s views and experiences of alcohol’s role in men’s violence to them.	Grounded theory adapted to suit feminist research principles	20 women who had been involved in an incident dealt with by the police domestic assault unit in Kingston-Upon-Hull	Participants reported alcohol’s role as initially positive in partner opening up but leading to violent and aggressive behaviour; partner’s capacity to drink; some women believed they provoked his violence by answering back, especially when they had been drinking; seven of nine believed alcohol played key role in their partner’s violence, but additional factors were considered to play a role in the violence. Proposed theory of ‘responsible disinhibition.’
Guggisberg [54]	Australia	To understand the nature of violence experienced by a current or former intimate partner	Phenomenological approach	3 Aboriginal women (mean age = 32.8 years)	Partners’ drinking was implicated in economic abuse and severity of abuse and sexual violence victimization. Women faced demands for their money at payday to buy alcohol; women felt intimidated and scared when their partner drank alcohol and then gets angry; fear from threats of being hurt with objects or weapons. Women’s readiness to retaliate was influenced by their own intoxication.
Hellum et al. [33]	Denmark	To investigate the difficulties, prior to seeking professional help, of female ‘concerned significant others’ of people with alcohol problems; and what led them to seek professional help.	Interpretative phenomenological	12 female help-seeking concerned significant others (CSOs) of men with alcohol problems recruited through RCT CRAFT study.	Participants reported high impacts on their physical and mental health, and quality of life, from a significant other’s drinking. These impacts stemmed from experiencing physical violence and mental abuse and included severe stress, social impacts, increased burden of care, loneliness and embarrassment, worry about children, and worry for the drinker themselves.
Kaur & Garg [44]	India	To understand the factors that perpetuate domestic violence and approaches to address it as felt by the women themselves.	Qualitative descriptive study	8 focus group discussions (FGD) with 72 married women 18–35 (8 to 10 women per FGD) in rural village, and 10 key informant interviews	IPV was commonly experienced by women in the focus groups (61%) and was more common for lower caste women. Almost 100% of the women blamed alcohol/alcoholism for IPV, although 20% also said violence sometimes occurs when their husband was not drinking. Some saw the violence as socially learned behaviour aiming at control.
Keenan et al. [48]	Lebanon and Egypt	To identify patterns of wife and child abuse	Qualitative content analysis of interview narratives	60 low-income women victims of abuse from Middle Eastern families living in Lebanon and Egypt; recruited from maternal child health settings	Small portion of the sample experienced abuse when their husband was drinking. Substance abuse was identified as one cause of physical abuse. Violence related to substance abuse was dramatic and severe. Beatings often occurred after challenge to husband’s authority by woman’s disapproving actions or words.
Kohli et al. [43]	Democratic Republic of Congo (DRC)	Study aims: 1) to describe the social and behavioral risk factors for IPV perpetration and victimization; 2) to describe the multiple and interrelated social, health and economic consequences of IPV on women and their families; and 3) to describe family and community driven response to IPV in rural villages	Grounded theory study	Five male IPV perpetrators and 13 female survivors who are participants in Pigs for Peace microfinance program in 3 villages in eastern DRC	Theme: Social and behavioral circumstances that increase risk for IPV. Male alcohol use was described as a direct ‘cause’ of IPV harms and indirectly through increased marital conflict. Study noted the role of male peer influence encouraging alcohol use leading to increased conflict in family and contributing to attitudes towards authority of male and subjugation of women. All participants highlighted the importance of male alcohol use, including excessive consumption, lack of employment, household financial stress, male infidelity and fear of female infidelity as a consequence of instability in rural communities and conflict and directly related to male use of physical and psychological violence. Among conflict-affected populations, elevated alcohol use was observed to deal with the prolonged stress and trauma.
Kyriakakis et al. [56]	USA	To gain a greater understanding of what Mexican immigrant women consider abusive in an intimate partner relationship, the abusive tactics employed and what abusive behaviour they found most hurtful	Phenomenological approach	29 Mexican immigrant women living in New York and St. Louis	Alcohol use was inextricably linked to husband’s abusive behaviour. More than two thirds of women had partners who drank heavily. There was no consistent pattern between husband’s level of intoxication and severity of violence. Participants reported subject to economic abuse, fear, infidelity, physical abuse, and brandishing of weapons linked to alcohol use. Participants viewed alcohol treatment as a solution for the abuse. However, partners were reluctant to seek treatment. Alcohol use was normalised.
Lennon et al. [53]	Colombia	To investigate factors that influence IPV against women.	Qualitative focus groups and interviews	28 female survivors of IPV and 15 key informants (professionals from psychological, social and legal services and community leaders)	Alcohol was one of four factors associated with IPV and was a big problem in the study population. Women associated their partner’s drinking with jealousy. Women explained that the primary reason for physical abuse was because he was drunk. The second reason was that he was jealous.
Mazibuko & Umejesi [34]	South Africa	To examine women’s narrative on alcohol consumption as the cause of domestic violence in relationships	Qualitative	27 survivors of domestic violence residing in a low-income black neighbourhood of Mamelodi Township, Pretoria	Common view from women was that alcohol is to blame for domestic violence; violence is driven by unseen external forces – the ‘devil.’ Women reported having to comply with their husbands’ sexual demands when he was drunk. Women experienced alcohol-related physical abuse; verbal and emotional abuse; social harm (embarrassment at their workplace); sexual coercion and abuse; and financial abuse (husband spending household income on alcohol).
Nascimento et al. [35]	Brazil	To know the daily life of women with alcoholic companions and their care options	Thematic oral history; interpretation guided by King’s Theory of Goal Attainment	11 women with alcoholic companions registered at a family health unit in Mato Grasso province, aged 18–45 years	Participants reported a range of impacts from their partner’s drinking including health harms from physical violence, fatigue, sleeplessness; mental harms from fear, instability, embarrassment and guilt, and anxiety over impacts on children including loss of a ‘father figure’; financial difficulties; and social and personal harms from loss of freedom, giving up on their life plans, and loss of motivation for self-care.
Rao [45]	India	To study the determinants of wife abuse	Ethnographic and econometric methods	Interviews with 70 women and 30 men across 3 villages in community of potters in Southern India; 14 focus group discussions	Determinants of domestic violence were excessive liquor consumption (mostly by men) and perceived inadequacy of dowries. Drunkenness was sometimes a catalyst for arguments becoming violent; other times drunken husbands assaulted partners without any provocation.
Satyanarayana et al. [36]	South India	To explore the intersection among alcohol consumption, gender roles, intimate partner violence and mental health from the perspective of heavy drinking men who also perpetrate IPV and their spouses (survivors)	Phenomenological approach – in-depth interviews	10 married heavy drinking men perpetrators and 10 IPV survivors (married spouse – currently cohabiting); not same dyad	Both perpetrators and survivors viewed drinking as the ‘root cause of all evil.’ Perpetrators attributed their aggression, violence and related behaviour to alcohol. Survivors reported depletion of household financial resources and increased debt due to spending on alcohol. Survivors reported sleep disturbances, body aches and pains, tiredness, not eating regular meals, losing weight and death wishes, shame, embarrassment, social estrangement and isolation (avoiding social situations due to humiliation from partner’s drinking and abuse).
Sedziafa et al. [49]	Ghana	To explore the health effects of IPV among 15 ever-partnered Ghanaian patrilineal women	In-depth interviews	15 ever-partnered Ghanaian patrilineal women aged between 25–65 years	Alcohol was viewed as the cause of male partner’s abusive attitudes. Women experienced physical violence and coerced sex when the husband was drunk.
Sørensen et al. [50]	Sri Lanka	To investigate daily life stressors in marriages and intimate relationships focusing on alcohol use and events of self-harm	In-depth narrative life story interviews and focus group discussions	44 participants aged 19–69 years (who had experienced alcohol-related events of self-harm and their family members)	Alcohol-related daily life stressors aggravated non-alcohol-related stressors (e.g. violence and financial difficulties) which contributed to partner conflict and episodes of self-harm. Alcohol aggravated overall levels of stress. Women described self-harm in response to stressors including husband alcohol use and gendered marriage expectations. Husbands’ alcohol use was shameful for wives. Alcohol often involved in domestic violence. Gendered alcohol norms were considered one of several acceptable excuses for the husband’s inappropriate behaviour.
Stockl & Gardner [55]	Germany	To investigate how abused women perceive their pregnancy to influence the context in which IPV occurs and the mechanisms leading to IPV	In-depth, semi-structured interviews	19 women who experienced violence during pregnancy	Participants with a heavy drinking partner reported loneliness as pregnancy restricted women from joining their partner in socialising. Home life was impacted as the expectations of the partner to stay home to support her during pregnancy were not met.
Tamutiene & Laslett [37]	Lithuania	To identify and contextualize the harms Lithuanian families experience when they include a heavy drinker	Grounded theory (Glaser and Strauss, 2009) analysis of in-depth, semi-structured interviews	20 women and 4 men cohabiting spouses and ex-partners of heavy drinkers purposively recruited via snowballing from Al-Anon and AA members and contacts	Direct harms caused by the drinker included: physical violence (including towards children); emotional abuse – disrespect, humiliation, contempt; financial abuse and debt. There was indirect harm to children from witnessing harm inflicted on others. Family functioning was affected by abdication or redirection of drinker’s responsibilities to other family members; care burden – care of the intoxicated drinker after heavy drinking, and care of family matters. Drinker-centred coping strategies neglected children’s (and other adults’) needs.
Wechsberg et al. [52]	South Africa	To investigate how alcohol and other drug use, gender power and gender violence are related to sexual risks for HIV among couples in established heterosexual relationships.	Content analysis	10 focus groups with men and women recruited from local taverns (67 participants) in two communities. Focus groups comprised women-only, men-only and couples.	Relationships issues were precursors to conflict and violence including disagreements over drinking and spending money. Discrepant drinking between the couple (both male and female) led to verbal arguments. Alcohol and other drug purchases affected financial well-being of household with males not providing for women and children. Men physically abused female partners because of lack of money for alcohol and/or other drugs and traded female partners for alcohol and other drugs. Women were forced to engage in ‘survival sex’ (prostitution) to make money for the household. Women had little condom negotiating power as drinking impaired men’s judgment around condom use.
Wilson et al. [21]	Australia	To explore the dynamics of drinking and IPV from the perspectives of women with lived experience of alcohol-related IPV	Grounded theory	18 women (20–50 years) who had experienced fear or harm from an alcohol-affected male partner	Findings describe a cycle of drinking and IPV and women’s safety strategies. Participants experienced the following related to partner’s alcohol use: sexual coercion/pressure; fear, threats, verbal abuse; humiliation; prioritising household spending on drinking, controlling finances, threatened/coerced to buy alcohol for partner; social restriction (work activities, contact with friends and family); jealousy, monitoring movements, control
Wilson et al. [22]	Australia	To understand the evolution of relationships involving alcohol-related IPV and how women identify and adapt to the changing nature of a partner’s drinking and violence	Grounded theory	18 women (20–50 years) who had experienced fear or harm from an alcohol-affected male partner	Findings describe processes engaged in by women to respond to and manage male partner’s alcohol-related IPV over key relationship stages – early relationship; committed relationship; relationship end, and beyond the abusive relationship. Women experienced physical, financial, social and emotional harms associated with partner drinking.
Wilson [38]	Australia	To examine the personal impacts of partner problem drinking and alcohol and other drug (AOD) use on individuals	Thematic analysis	100 partner transcripts from anonymous online chat counselling service for problematic AOD use extracted between 2013–2014 (females 85% of partners; males 15%)	Themes: (1) cognitions (depressive cognitions, responsibility beliefs, and thoughts around trust); (2) behaviours (helpful and unhelpful coping) and (3) emotions (anger, sadness, and fear).
Wilson et al. [39]	Australia	To examine the broad range of interpersonal impacts described by partners from online counselling transcripts sourced from a national online AOD counselling service	Thematic analysis	100 partner transcripts from anonymous online chat counselling service for problematic AOD use extracted between 2013–2014 (females 85% of partners; males 15%)	Themes: (1) Intimate partner relationship issues, (2) parenting issues, (3) impacts on/from partners’ social networks arising from partners’ drinking. Communication problems and conflict centred around problem AOD use, escalated to verbal and physical confrontations. AOD use impacted quality time in relationships. Concerns about impact on children of exposure to AOD problem use, parental conflict and partner abuse.
